# The impact of interior dielectric constant and entropic change on HIV-1 complex binding free energy prediction

**DOI:** 10.1063/1.5058172

**Published:** 2018-12-14

**Authors:** Yuchen Li, Yalong Cong, Guoqiang Feng, Susu Zhong, John Z. H. Zhang, Huiyong Sun, Lili Duan

**Affiliations:** 1 School of Physics and Electronics, Shandong Normal University, Jinan 250014, China; 2 Shanghai Engineering Research Center of Molecular Therapeutics and New Drug Development, School of Chemistry and Molecular Engineering, East China Normal University, Shanghai 200062, China; 3 NYU-ECNU Center for Computational Chemistry at NYU Shanghai, Shanghai 200062, China; 4 Department of Chemistry, New York University, New York, New York 10003, USA; 5 Department of Medicinal Chemistry, School of Pharmacy, State Key Laboratory of Natural Medicines, China Pharmaceutical University, Nanjing 210009, China

## Abstract

At present, the calculated binding free energy obtained using the molecular mechanics/Poisson-Boltzmann (Generalized-Born) surface area (MM/PB(GB)SA) method is overestimated due to the lack of knowledge of suitable interior dielectric constants in the simulation on the interaction of Human Immunodeficiency Virus (HIV-1) protease systems with inhibitors. Therefore, the impact of different values of the interior dielectric constant and the entropic contribution when using the MM/PB(GB)SA method to calculate the binding free energy was systemically evaluated. Our results show that the use of higher interior dielectric constants (1.4–2.0) can clearly improve the predictive accuracy of the MM/PBSA and MM/GBSA methods, and computational errors are significantly reduced by including the effects of electronic polarization and using a new highly efficient interaction entropy (IE) method to calculate the entropic contribution. The suitable range for the interior dielectric constant is 1.4–1.6 for the MM/PBSA method; within this range, the correlation coefficient fluctuates around 0.84, and the mean absolute error fluctuates around 2 kcal/mol. Similarly, an interior dielectric constant of 1.8–2.0 produces a correlation coefficient of approximately 0.76 when using the MM/GBSA method. In addition, the entropic contribution of each individual residue was further calculated using the IE method to predict hot-spot residues, and the detailed binding mechanisms underlying the interactions of the HIV-1 protease, its inhibitors, and bridging water molecules were investigated. In this study, the use of a higher interior dielectric constant and the IE method can improve the calculation accuracy of the HIV-1 system.

## I. INTRODUCTION

Molecular dynamics (MD) simulation is a popular and efficient tool that has been widely employed to study protein-protein binding, protein-inhibitor interactions, and protein structure, dynamics, and folding at the atomic level.^1,2^ The accuracy of MD simulations depends mainly on the accuracy of the force field that is used. The results of MD simulations based on inaccurate force fields may lead to the lack of accuracy and reliability in computational studies of biological systems. As we know, electrostatic interactions play a significant role in protein-inhibitor interactions. However, in the current standard force fields that are used, such as AMBER, CHARMM, and OPLS, electrostatic interactions are described using fixed point charge interactions, without taking into account the polarization effect of proteins.^3,4^ Some polarizable force fields are developed to overcome this deficiency, such as the fluctuating charge model,^5^ Drude oscillator,^6^ and induced multi-pole.^7^ But the practical applications of these models are uncommon because of the limitations such as high computational costs and the validity of the results. To obtain a more reliable description of the electrostatic interactions, the polarized protein-specific charge (PPC) force field^8–11^ is used in our study. PPC is derived from quantum mechanical calculations that are applied to proteins in solution using the molecular fractionation with conjugate cap (MFCC) approach,^12^ and the effects of the solvent surrounding the proteins are determined using the Poisson-Boltzmann (PB) equation. Previous studies showed that the PPC force field can successfully fold the helix protein, while the AMBER02 polarizable force field failed. We also compared the dynamical property between the PPC force field and two versions AMBER02 and AMBER12 polarizable force fields and found that the PPC force field gave more stable structures than the other two polarizable force fields.^13,14^ This method successfully incorporates electrostatic polarization effects, and its advantages over the traditional force field have been shown in a series of studies.^8–11,15–18^

Accurate computation of the free energy of protein-inhibitor binding is very important in clarifying the structure-affinity relationships and investigating the binding mechanisms that are involved. On the one hand, it is necessary to ensure the accuracy of the force field that is used and obtain abundant ensemble sampling during MD simulation; on the other hand, the highly efficient and reliable computation of the entropic change is also critical to the accuracy of the binding free energy calculation. To date, a number of methods have been used to calculate the binding free energy, such as the free energy perturbation (FEP),^19–24^ thermodynamic integration (TI),^25,26^ and molecular mechanics/Poisson-Boltzmann (Generalized-Born) surface area (MM/PB(GB)SA) methods.^27–29^ In general, the FEP and TI methods are considered to be the most rigorous approaches for the calculation of the binding free energy. However, both these methods have flaws due to time-consumption and the limitations in the calculation of the relative free energy. In contrast, the MM/PBSA method, which is widely employed to calculate the absolute binding free energy, has become a popular method used to elucidate the mechanism of interaction between proteins and inhibitors.^30–36^

According to the MM/PBSA method, the entropic contribution is usually evaluated using the standard normal mode (Nmode) method, which is extremely expensive and is approximate, in theory.^37^ The Nmode method assumes that the superposition of vibrations with different frequencies can reflect the internal motions of protein, and then, the vibrational entropy can be calculated. The Nmode method decomposes the entropic contribution into three parts which are changes in translational, rotational, and vibrational freedoms. Due to the lack of a reliable and efficient method for calculating entropic changes, many previous studies neglected the entropic contribution to the binding free energy when applying the MM/PBSA method, which resulted in some inaccurate results.^37–45^ However, a number of methods have been developed to calculate entropic changes, such as that described by Swanson *et al.*, which is a novel method for calculating the entropic change arising from a single molecule's loss of translational and rotational freedom.^46^

Recently, a new entropy computation method, known as the interaction entropy (IE) method, has been developed by our group^47^ that is theoretically rigorous, computationally efficient, and numerically reliable. A recent study showed that IE produces a more reliable estimate of the entropic contribution of the interaction of protein-ligand, protein-protein, and hot-spot residues for use in calculating the binding free energy.^48–55^ Due to its simplicity and practicality, the IE method has been widely used by our own group as well as other researchers to calculate many different types of binding free energies and explain important interaction mechanisms in biological systems.^41,56–62^

Due to its lack of effect on protein motion and polarization, the interior dielectric constant (dielectric in the solute) is usually set to 1 when calculating the electrostatic energy (the Coulomb term in the force field) and polar solvation energy using the MM/PBSA method; this is imprecise,^40,63^ due to the fact that electrostatic interactions are not shielded enough and protein-inhibitor electrostatic attractions and repulsions are overestimated, leading to an inaccurate prediction.^63^ The protein-inhibitor binding free energy is quite sensitive to the interior dielectric constant,^40^ and it has been shown by some researchers that an interior dielectric constant of 1 is not appropriate for some systems.^48,55^ Thus, it is necessary to evaluate the impact of the interior dielectric constant on the calculation of the binding free energy and determine the appropriate interior dielectric constants for different types of protein-inhibitor complexes. The Hou group found that MM/PBSA (GBSA) produces better results with a higher dielectric constant, such as 2 or 4, in most cases;^64–66^ due to the higher computational costs of using the Nmode method, the entropic contribution was neglected during their studies. Upon testing various values of the dielectric constant, it was found that the calculation of the binding free energy of Human Immunodeficiency Virus (HIV-1) protease^67–69^ had much worse performance than that of other systems using different dielectric constants in combination with low correlation coefficients that were obtained experimentally.^65^ They attribute this to two factors: (1) the fact that the bridging water molecule WAT301, which forms four hydrogen bonds with the protease and the inhibitor and has a significant impact on the binding free energy calculation, is not considered in their study, and (2) the entropic contribution, which has an important effect on the prediction of the binding free energy, is also ignored because of the higher computational cost of using the Nmode method. There are also other issues in the calculation of the HIV-1 protease-inhibitor binding free energy using the MM/PB(GB)SA method. The Wilson group examined the ability of the MM/PBSA and MM/GBSA methods to rank the inhibitors of HIV-1 protease, and they found that the binding effectiveness of larger inhibitors was overestimated by the MM/PB(GB)SA method and the correlation of the calculated values with the experimental values was very low, with a magnitude of less than 0.1 for MM/PBSA and 0.50–0.60 for MM/GBSA.^70^ The Coveney group also found that MM/PB(GB)SA overestimated the binding efficiency of larger inhibitors of HIV-1, resulting in a poor ranking ability. In addition, the consideration of the free energy of association had no effect on the ranking ability of MM/PBSA while resulting in only a slight improvement for MM/GBSA.^71,72^

In this paper, we set the solute dielectric constant to 1–2 with an interval between 0.1 and 4 for the binding free energies predicted using the MM/PBSA (GBSA) method for 10 different HIV-1 protease systems. The Nmode and IE methods are used to compute the entropic contribution, and the contribution of the bridging water molecule is also considered in our study.

## II. METHOD

### A. Molecular dynamics simulation

**FIG. 1.**
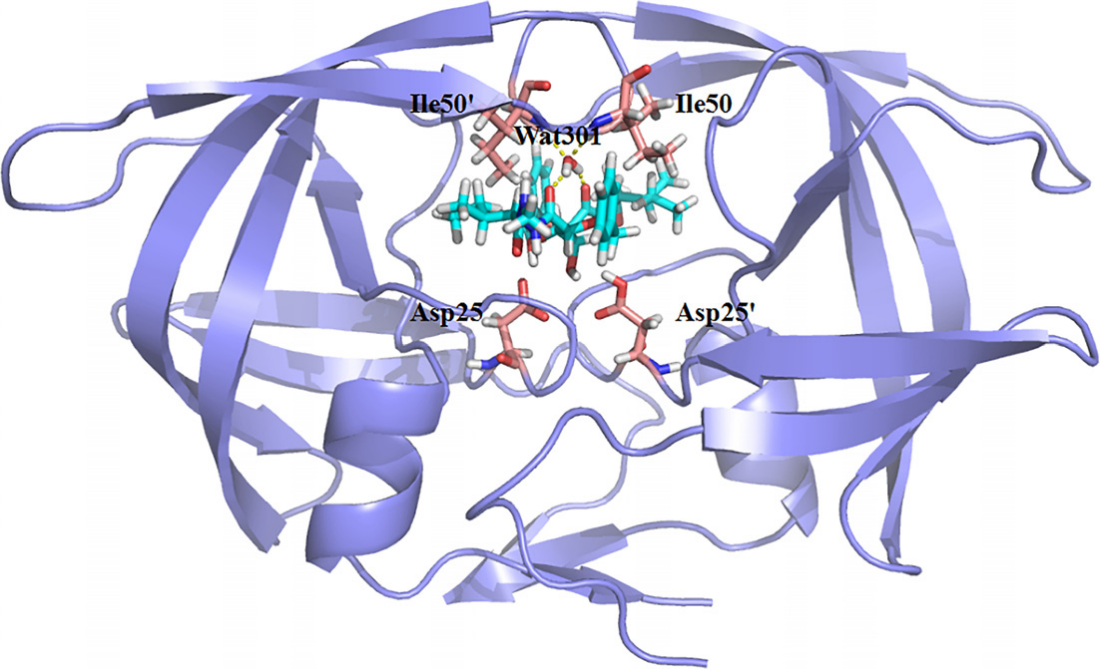
The native structure of HIV-1 protease with inhibitor.

Due to the inaccuracy of the binding free energy calculated for the charged ligand using MM/PBSA,^65^ we randomly selected ten protein-ligand systems with an uncharged ligand and known experimental binding energy values that spanned a range of 6 kcal/mol from the Protein Data Bank (PDB) bind database that was developed by Wang *et al.*^73^ In addition, the bridging water molecule was included in these selected systems. The ten initial complexes of the HIV-1 protease and various inhibitors were obtained from the Protein Data Bank (PDB entries 1BDR, 2BQV, 2CEN, 1W5Y, 1EC0, 2CEJ, 1EBW, 1C70, 2HB3, and 2I4U). The native structure of HIV-1 protease with inhibitor (PDB: 1EBW) is displayed in Fig. 1. All the missing hydrogen atoms in each complex were added using the Leap module in AMBER14.^74^ Due to the fact that the bridging water molecule, WAT301, forms four hydrogen bonds with the protease and the inhibitor, it was retained in the initial model. In addition, the OD2 of residue ASP25 in the A chain was protonated, based on the results of a previous study.^75,76^ Prior to the MD simulations, all the inhibitors were optimized at a level of HF/6–31G** and then employed to calculate the single point energy at the level of B3LYP/cc-PVTZ to obtain the electrostatic potentials (ESPs) and the atomic charges, which were fitted using the restrained ESP (RESP) method.^77^ The AMBER12SB force field and the generalized Amber force field (GAFF) were used for the calculations for the protein and the ligand, respectively. Each system was placed within TIP3P water molecules inside a truncated octahedron periodic box. The distance between the edges of the box and the closest solute atoms was 12 Å. Counter-ions were added to preserve the electrical neutrality of the system. The system was then optimized using the steepest descent method followed by conjugate gradient minimization in two stages to remove poor contacts between the solute and solvent water molecules. First, the water molecules were optimized by fixing the solute with an external force. Second, the entire system was energy-minimized without any constraints. Next, the entire system was heated from 0 to 300 K for 300 ps with a weak restraint of 10 kcal/(mol Å^2^)^−1^ for the complex, and Langevin dynamics was employed to regulate the temperature at a collision frequency of 1.0 ps^−1^. The time-step was set to 2 fs, and SHAKE was used to constrain the bonds involving the hydrogen atom.^78^ Finally, 10 ns of MD simulation was carried out at 300 K. During the first 8 ns, the trajectories were saved every 1 ps. In the last 2 ns, 200 000 fames have been used to the binding free energy calculation. Therefore, numerous snapshots were extracted from the MD trajectories and used to calculate the entropic contribution using the IE method to obtain the convergent interaction energy and interaction entropy. Sampling of at least 200 000 frames was essential for improving the accuracy of the binding free energy calculation.

### B. Polarized protein-specific charge

Polarized Protein-specific Charge (PPC)^8^ is generated by applying the molecular fragmentation with conjugate caps (MFCCs),^12^ in which the quantum mechanical (QM) calculation of protein is made possible, incorporating the Poisson-Boltzmann (PB) solvation model. The basis procedures of fitting atomic charge are as follows: at first, the MFCC scheme is used to decompose the entire protein into amino acid to obtain the electronic density distribution of the fragment molecules and inhibitor through fully QM calculations. Then, the partial charge of each atom and inhibitor is fitted by using the RESP program^77^ according to the electron density distribution of each fragmental molecular. Next, the PB equation is used to solve the self-consistent reaction field to obtain the induced charges on the complex-solvent interface. The induced charges mimicked the solvation effect, and the newly obtained atomic charges of other residues are used as background charges in the calculation of the next cycle QM calculation to fit new partial charges. Finally, the solute and solvent polarize each other until solvation energy and induced charges have converged. The new polarized protein-specific charges are obtained and the solute charges are replaced by the PPC when performing MD simulation using the PPC force field.

### C. MM/PBSA (GBSA) method

**FIG. 2.**
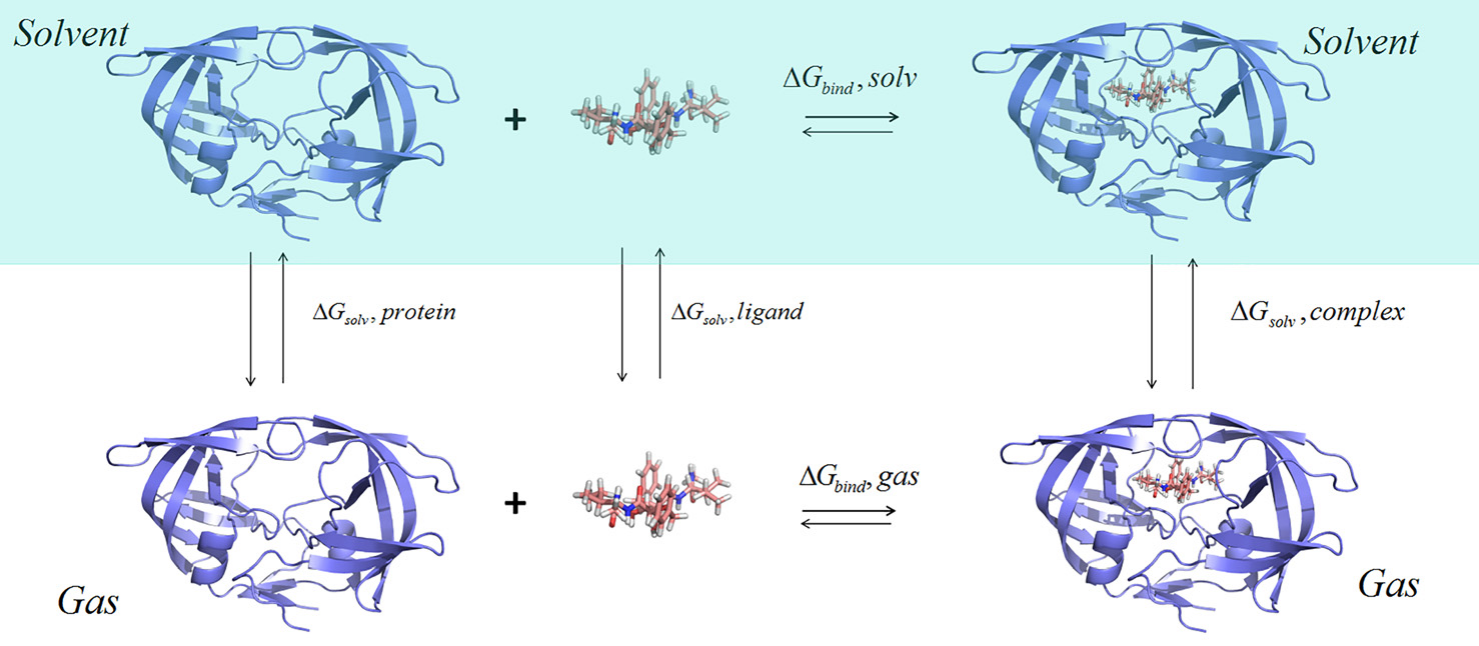
The thermodynamics cycle of the MM/PBSA (GBSA) method.

In this work, the MM/PBSA (GBSA) method is used to calculate the binding free energy of HIV-1 protein-inhibitor complexes. The thermodynamics cycle is displayed in Fig. 2. The MM/PBSA (GBSA) method can be described by the following form:

$$\Delta G_{\text{bind}}=G_{\text{complex}}-(G_{\text{protein}}+G_{\text{ligand}}),$$ (1)

where $G_{\text{complex}}$, $G_{\text{protein}}$, and $G_{\text{ligand}}$ represent the free energy of the protease-inhibitor complex, protease, and inhibitor, respectively. In addition, the binding free energy ($\Delta G_{\text{bind}}$) contains the gas-phase binding free energy ($\Delta G_{gas}$) and the solvation free energy of the complex relative to that of the protein and inhibitor ($\Delta \Delta G_{sol}$)

$$\Delta G_{\text{bind}}=\Delta G_{gas}+\Delta \Delta G_{sol}.$$ (2)

Gas-phase binding free energy ($\Delta G_{gas}$) can be divided into the ensemble-averaged interaction of protease-inhibitor ($\left\langle E_{pl}^{\mathrm{int}}\right\rangle$), which includes electrostatic interaction and van der Waals (vdW) interaction, and the contribution of entropy (−TΔS)

$$\Delta G_{gas}=\langle E_{pl}^{\mathrm{int}}\rangle -T\Delta S.$$ (3)

$\Delta \Delta G_{sol}$ can be divided into two parts

$$\Delta \Delta G_{sol}=\Delta \Delta G_{pb/gb}+\Delta \Delta G_{np},$$ (4)

where $\Delta \Delta G_{pb/gb}$ and $\Delta \Delta G_{np}$ represent the polar and non-polar terms, respectively. The term $\Delta \Delta G_{pb/gb}$ can be computed using the PB and GB module of the MM/PBSA (GBSA) method. In this study, the exterior dielectric constant is set to 80 and the interior dielectric constant is set to 1–2 with the interval of 0.1 and 4 for electrostatic interaction and solvation energy computation, respectively. The term $\Delta \Delta G_{np}$ is based on the following equation:

$$\Delta \Delta G_{np}=\gamma \cdot \text{SASA}+\beta ,$$ (5)

where *SASA* represents the solvent-accessible surface area, and it can be calculated using the MSMS program.^79^ The numerical values of γ and β are set to the standard values of 0.00542 kcal (mol Å^2^)^−1^ and 0.92 kcal mol^−1^,^80^ respectively.

Finally, the Nmode module^81^ is used to calculate the entropic contribution using the following form:

$$\Delta S_{NM}=\Delta S_{tra}+\Delta S_{rot}+\Delta S_{vib},$$ (6)

where $\Delta S_{tra}$, $\Delta S_{rot}$, and $\Delta S_{vib}$ are the energy contributions related to changes in translational, rotational, and vibrational freedoms, respectively.

Due to the huge expensive computational cost for large systems, only 10 snapshots from the last 2 ns MD trajectory are selected using the Nmode method.

In addition, the bridging water WAT301 is considered as a part of the protease in the binding free energy calculation.

### D. Interaction Entropy (IE) method

The entropic contribution can be defined according to the following formula:^47^

$$\Delta G_{\text{gas}}=-KT\;\text{ln}\;\frac{\int dq_{w}dq_{p}dq_{l}e^{-\beta (E_{p}+E_{l}+E_{pl}^{\mathrm{int}}+E_{w}+E_{pw}^{\mathrm{int}}+E_{lw}^{\mathrm{int}})}}{\int dq_{w}dq_{p}dq_{l}e^{-\beta (E_{p}+E_{l}+E_{w}+E_{pw}^{\mathrm{int}}+E_{lw}^{\mathrm{int}})}}$$

$$=-KT\left[\frac{1}{\left\langle e^{\beta E_{pl}^{\mathrm{int}}}\right\rangle }\right]$$

$$=KT\langle e^{\beta E_{pl}^{\mathrm{int}}}\rangle$$

$$=\langle E_{pl}^{\mathrm{int}}\rangle +KT\;\text{ln}\;\langle e^{\beta \Delta E_{pl}^{\mathrm{int}}}\rangle ,$$ (7)

$$\Delta G_{gas}=\langle E_{pl}^{\mathrm{int}}\rangle -T\Delta S,$$ (8)

$$-T\Delta S=KT\;\text{ln}\;\langle e^{\beta \Delta E_{pl}^{\mathrm{int}}}\rangle ,$$ (9)

where $\Delta E_{pl}^{\mathrm{int}}$ represents the fluctuation of the protease-inhibitor interaction energy in relation to the average interaction energy, which can be calculated using the following formula:

$$\Delta E_{pl}^{\mathrm{int}}=E_{pl}^{\mathrm{int}}-\langle E_{pl}^{\mathrm{int}}\rangle ,$$ (10)

where $\left\langle E_{pl}^{\mathrm{int}}\right\rangle$ represents the average of the protein-inhibitor interaction energies and $E_{pl}^{\mathrm{int}}$ is the interaction energy between a protease and an inhibitor.

Using the IE method, $\left\langle E_{pl}^{\mathrm{int}}\right\rangle$ and $\left\langle e^{\beta \Delta E_{pl}^{\mathrm{int}}}\right\rangle$ can be conveniently and efficiently computed using the following equations:

$$\langle E_{pl}^{\mathrm{int}}\rangle =\frac{1}{T}\int_{0}^{T}E_{pl}^{\mathrm{int}}\left(t\right)dt=\frac{1}{N}\sum_{i=1}^{N}E_{pl}^{\mathrm{int}}\left(t_{i}\right)$$ (11)

and

$$\langle e^{\beta \Delta E_{pl}^{\mathrm{int}}}\rangle =\frac{1}{N}\sum_{i=1}^{N}e^{\beta \Delta E_{pl}^{\mathrm{int}}\left(t_{i}\right)},$$ (12)

where β represents $\frac{1}{KT}$.

As can be seen, the interaction energy between the protease and the inhibitor is an important component of the entropic change computation in the IE method. As long as the protease-inhibitor interaction energy is obtainable, the entropic contribution can be calculated simply, directly, and efficiently.

It should be noted that the entropic change before and after the binding of the protein and the ligand can be described by the following equation:

$${-T\Delta S}_{tot}={-T\Delta S}_{pl}{-T\Delta S}_{int},$$ (13)

where ${-T\Delta S}_{pl}$ represents the entropic change between the protein and the ligand and ${-T\Delta S}_{int}$ represents the internal entropic change within the protein and ligand molecules. If the protein structure does not undergo a large conformational change before and after the binding of the ligand and the ligand is relatively rigid, the internal entropic change should be negligible. Thus, the interaction entropy is equivalent to the entropic change as determined from the rigid body approach without internal conformational and rotational entropic changes upon protein-ligand binding.

## III. RESULTS AND DISCUSSION

### A. The analysis of stability

In our study, two MD simulations were conducted using the AMBER and PPC force fields.

**FIG. 3.**
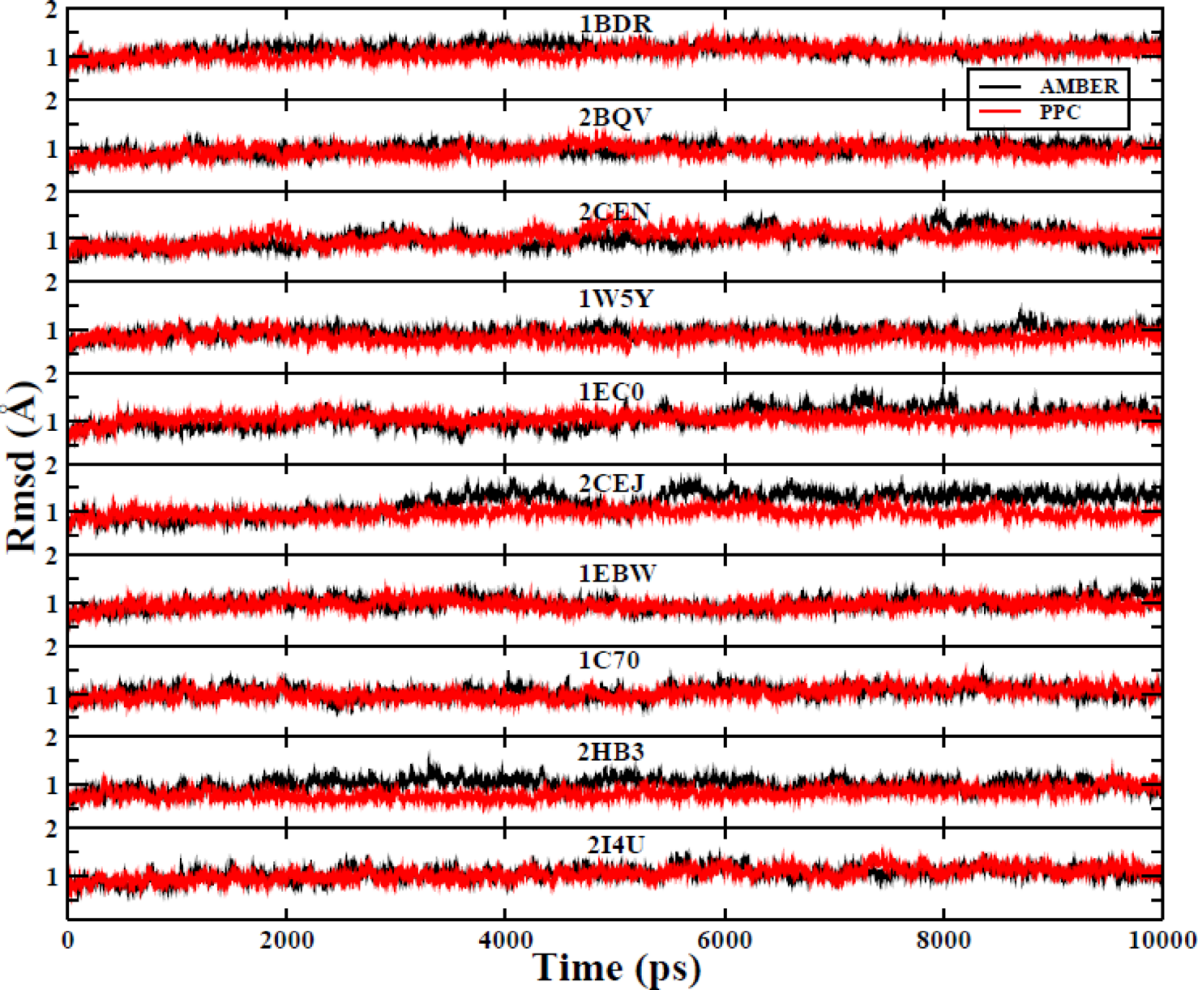
The ten systems' RMSD of the backbone atoms relative to the corresponding native structure during the whole MD simulation. The AMBER force field is black, and the PPC force field is red.

**FIG. 4.**
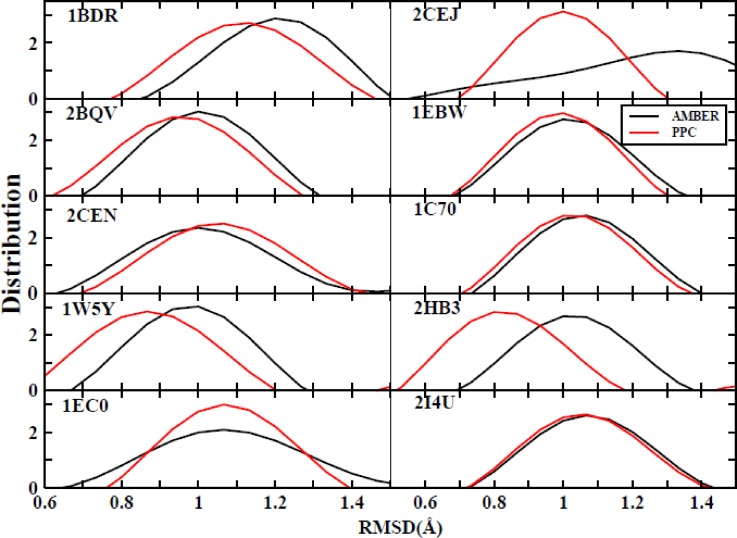
The distribution of the ten systems' RMSD. The AMBER force field is black, and the PPC force field is red.

In order to evaluate the stability of the MD simulations, the root mean square deviations (RMSDs) of the backbone atoms in each of the ten systems from those of their corresponding native structure as a function of the time of the MD simulation are shown in Fig. 3, with the distributions of the RMSDs for the AMBER and PPC force fields shown in Fig. 4. We first conducted 8 ns of simulation and found that most of the RMSD values fluctuated between 0.6 Å and 1.5 Å (Fig. 3). This indicates that most of the simulations reached equilibrium after approximately 8 ns in both the force fields. It should be noted, however, that there are no clear criteria by which it can be judged that a simulation has reached equilibrium. In general, if the RMSD is less than 2 Å and there is no large fluctuation, the simulation is considered to reach equilibrium. Therefore, we conducted an additional 2 ns of simulation to calculate the binding free energy. As shown in Fig. 4, it is apparent that RMSDs for most of the ten systems in the PPC force field were smaller than those in the AMBER force field, which indicates that the PPC force field can provide a reliable and dynamically stable structure. This result demonstrates the important effects of electronic polarization in MD simulations and indicates that the use of the PPC force field was appropriate for our subsequent experiments.

### B. The convergence of the interaction energy and entropy

**FIG. 5.**
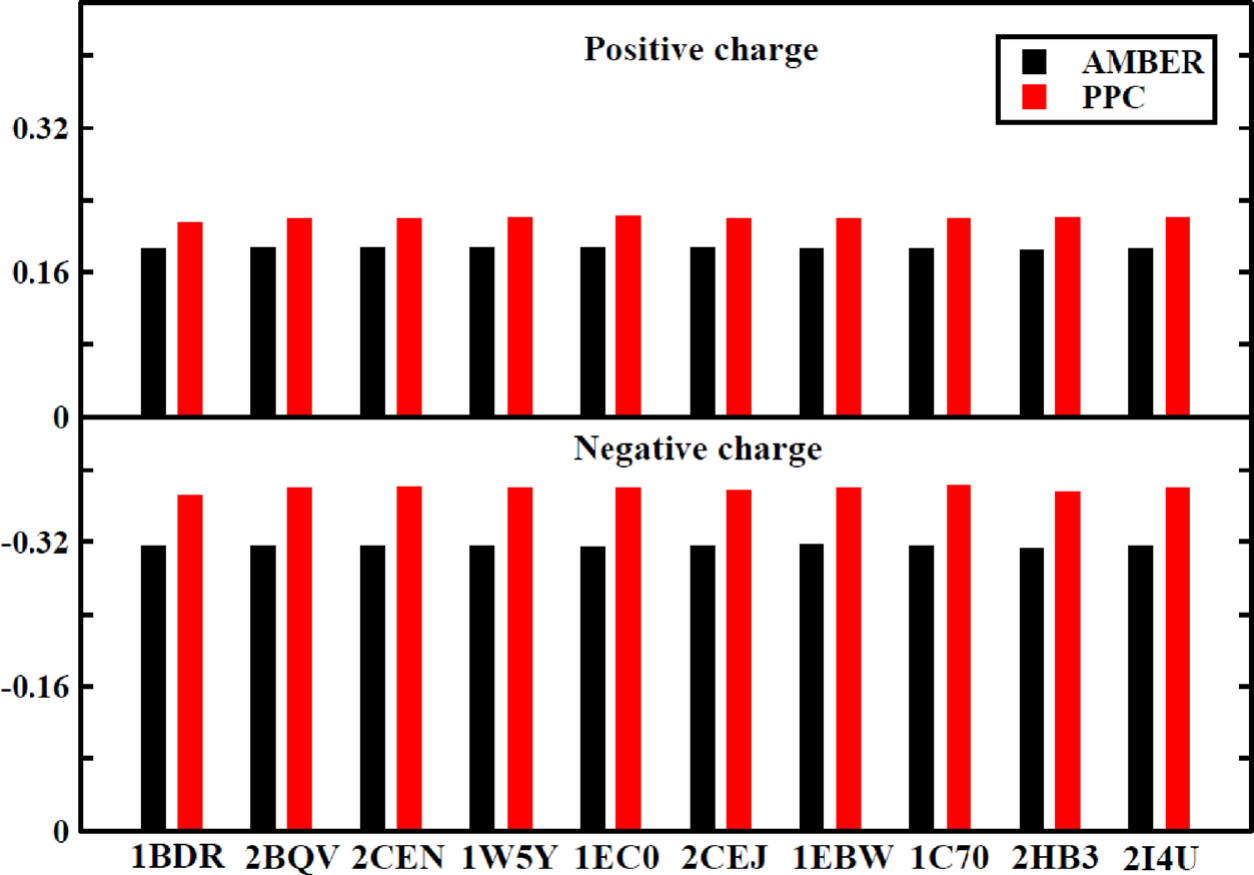
The average positive and negative charges of each system under the two force fields. The AMBER force field is black, and the PPC force field is red.

Before calculating the binding free energy, we first determined whether the interaction energy and interaction entropy had adequately converged for each of the ten systems. The fluctuation of the average interaction energy and interaction entropy in each of the ten systems during the last 2 ns of MD simulation for the two force fields are displayed in detail in Figs. S1 and S2. It can be seen that the calculated interaction energy and interaction entropy have reached convergence in both the force fields. We further estimated the performance of the interaction entropy and the standard deviations of the entropic change calculated using the IE and Nmode methods (Table S1). As shown in Table S1, the computational errors are significantly smaller for the IE method than the Nmode method for the ten tested systems, regardless of whether the AMBER or PPC force field was used. This suggests that the calculation of the entropic change using the IE method results in better convergence and greater statistical stability than the Nmode method. Furthermore, due to the disregard of the protein electronic polarization, the calculated interaction energies are consistently weaker in the AMBER force field than the PPC force field for the ten systems shown in Fig. S1. To account for the variation, we listed the average values of the positive and negative charges in each of the ten systems in Fig. 5. A clear difference can be seen, as the absolute values of the charges in PPC are larger than those in AMBER for the ten systems. The larger polarized charges produce greater electrostatic interaction in the PPC force field than in the AMBER force field, which results in stronger electrostatic interaction energy. These deviations illustrate that the inclusion of accurate estimates of the polarization effect in MD simulations can produce a stable and reliable electrostatic environment. The relatively weak energy resulted in some broken hydrogen bonds during MD simulations in the AMBER force field. The changes in the lengths of specific hydrogen bonds between the protease and inhibitor over time during MD simulations in both force fields are shown in Fig. S3. Some hydrogen bonds were broken or underwent large fluctuations during simulations in AMBER, while the same hydrogen bonds were well preserved during simulations in PPC. This phenomenon was also observed in many previous studies.^50,82–85^ The current study highlights the important effects of electrostatic polarization on MD simulation.

#### 1. The impact of the higher interior dielectric constant of electrostatic energy, polar solvation energy, and entropic contribution on the prediction of the binding free energy

In most previous studies, the interior dielectric constant (dielectric in the solute) was usually set to 1 when calculating the electrostatic energy and the polar solvation energy, which leads to the neglect of the effects of protein motion and polarization.^63^ In this case, the electrostatic interactions are not adequately shielded, which results in the overestimation of electrostatic attraction and repulsion between the protein and the inhibitor.^86,87^ The prediction of the protein-inhibitor binding free energy is quite sensitive to the interior dielectric constant; thus, it is necessary to evaluate the impact of the interior dielectric constant on the calculation of the binding free energy and determine suitable interior dielectric constants for the different types of protein-inhibitor complexes. Hou *et al.* used relatively high dielectric constants (ε = 2 or 4) to evaluate the impact of dielectric constants on the prediction of the binding free energy of more than 1800 protein-ligand complexes. Their studies found that the correlation coefficients obtained from the comparison of the calculated values with the experimental values were best for the MM/PBSA and MM/GBSA methods.^65,66^ However, their predictions for the HIV-1 protease system used in their study were poor, due to their neglect of the contribution of entropy to affinity.^65^ In order to investigate the impact of the interior dielectric constant on simulations of the HIV-1 system during this study, the interior dielectric constant was set to 1–2 with an interval of 0.1 and 4 for the computation of the electrostatic energy and the polar solvation energy, respectively.

**FIG. 6.**
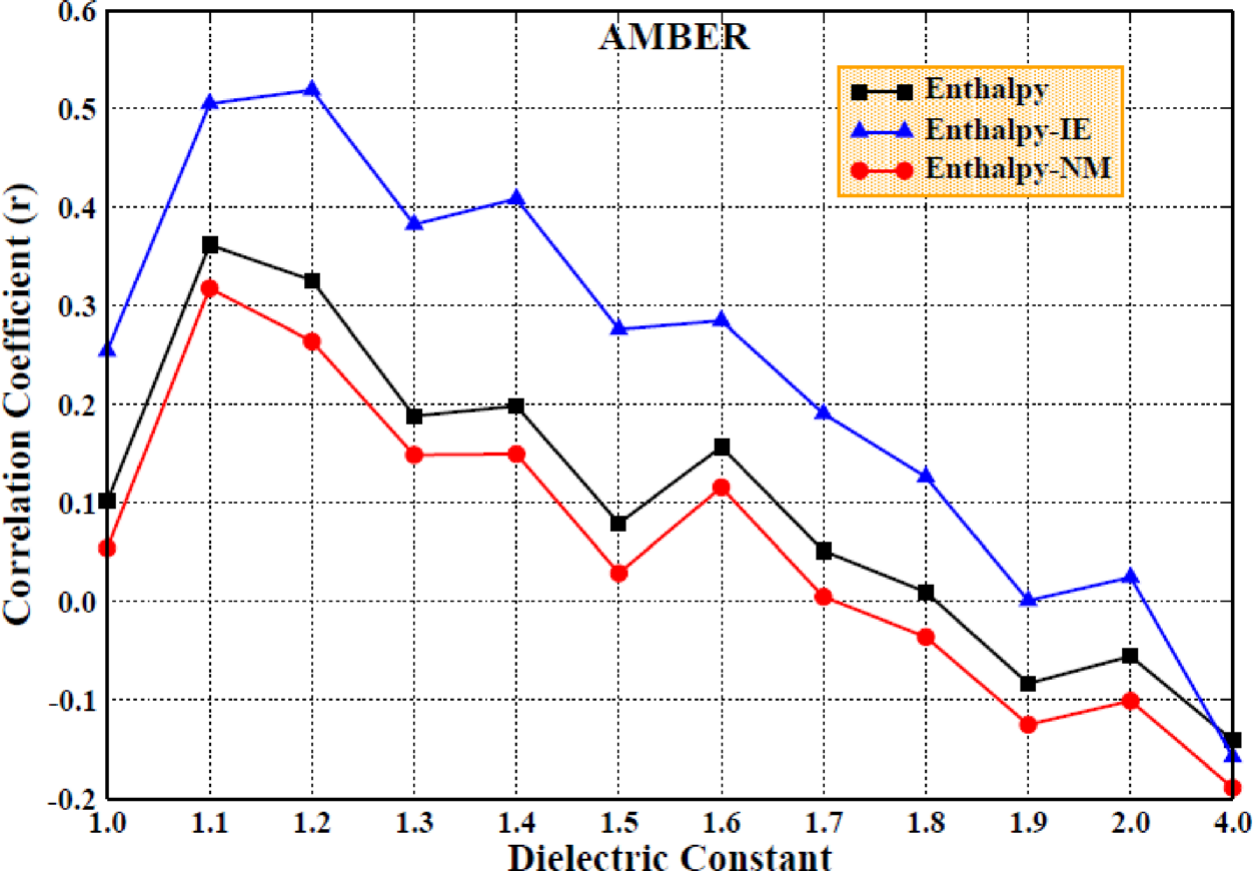
The correlation coefficients of enthalpy changes with experimental values (Enthalpy) and the correlation coefficients of the predicted binding free energy including entropic contribution computed by the Nmode/IE method with experimental values under the AMBER force field (Enthalpy-IE; Enthalpy-NM) (setting the interior dielectric constant to 1–2 with the interval of 0.1 and 4 for the electrostatic energy and polar solvation energy computation, respectively; polar solvation energy is computed by the MM/PBSA method).

**FIG. 7.**
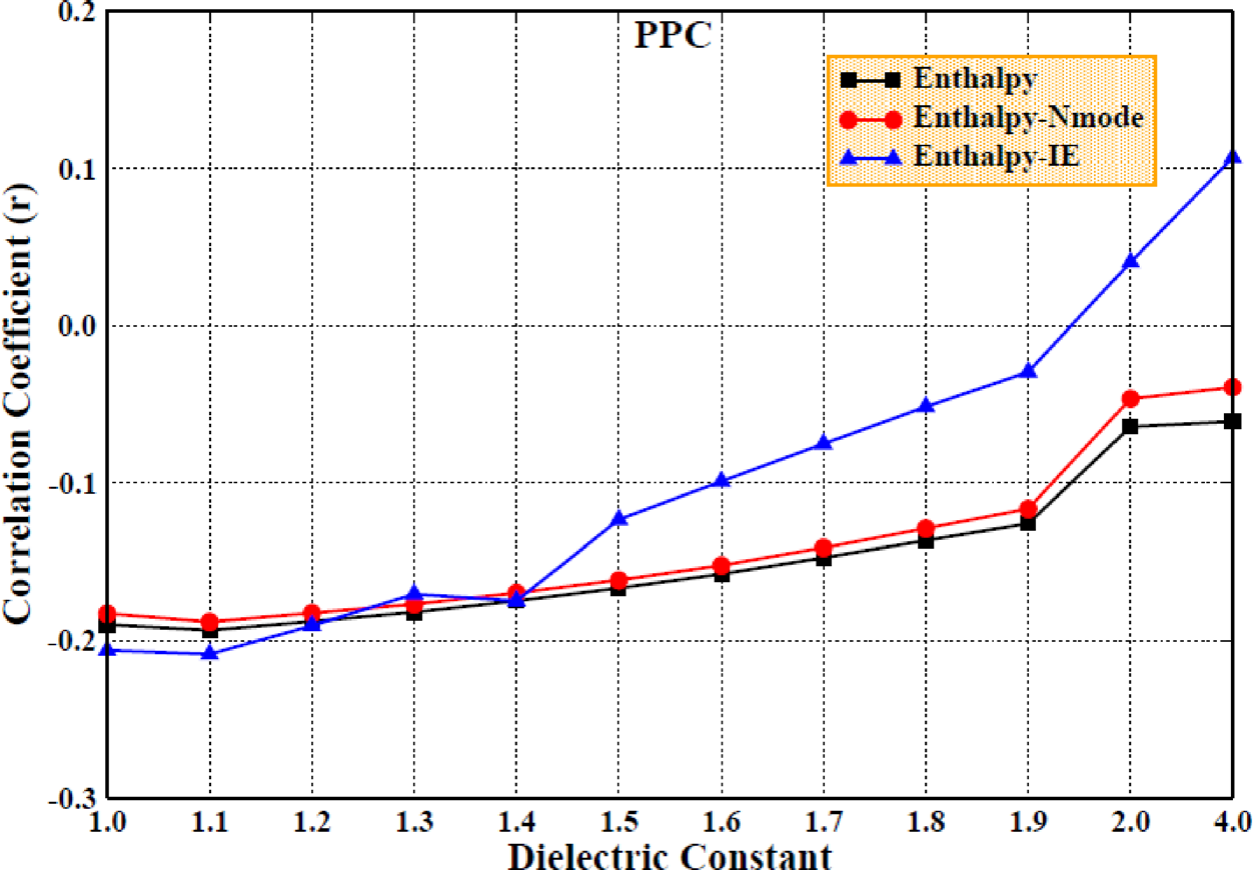
The same as Fig. 4, but in the PPC force field.

Due to the difficulty in calculating the entropic contribution, many studies ignored the entropic change and only consider the enthalpic contribution to the binding free energy, which may result in the inaccuracy and unreliability of the computational results. Therefore, we first determined the enthalpic contribution with a higher interior dielectric constant using the MM/PBSA method in both force fields. The correlation coefficients obtained for the comparison of the calculated and experimental values are shown in Figs. 6 and 7 for the AMBER and PPC force fields, respectively, which show poor linear relationships and are relatively low, regardless of the force field used.

We then included the entropy calculation in the determination of the binding free energy using the Nmode and IE methods; obviously, the correlation coefficients were improved, especially when using the highly efficient IE method. However, the results did not improve when using the Nmode method. In Fig. 7, it can be seen that the correlation coefficient improved slightly with the increase in the interior dielectric constant, which demonstrates that although a higher interior dielectric constant (1–2, with an interval of 0.1 and 4) and entropic contribution can improve the correlation coefficients, it still does not produce a significantly strong linear correlation between the calculated values and the experimental values.

Based on the above results, the use of a higher interior dielectric constant (1–2, with an interval of 0.1 and 4) for the electrostatic and polar solvation energy simultaneously may not be ideal for simulations of the HIV-1 system. Therefore, we only used a higher interior dielectric constant for electrostatic energy computation to examine its impact on the binding free energy calculation during subsequent experiments.

#### 2. The impact of the use of a higher interior dielectric constant for electrostatic energy computation and the entropic contribution of binding free energy on the performance of the MM/PBSA method

Because the calculated and experimental values obtained by simultaneously setting a higher interior dielectric constant for the computation of the electrostatic and polar solvation energy have not had a strong correlation, we set the interior dielectric constant to 1–2 with intervals of 0.1 and 4 for the computation of the electrostatic energy computation and to 1 for the computation of the polar solvation energy using the MM/PBSA method.

**FIG. 8.**
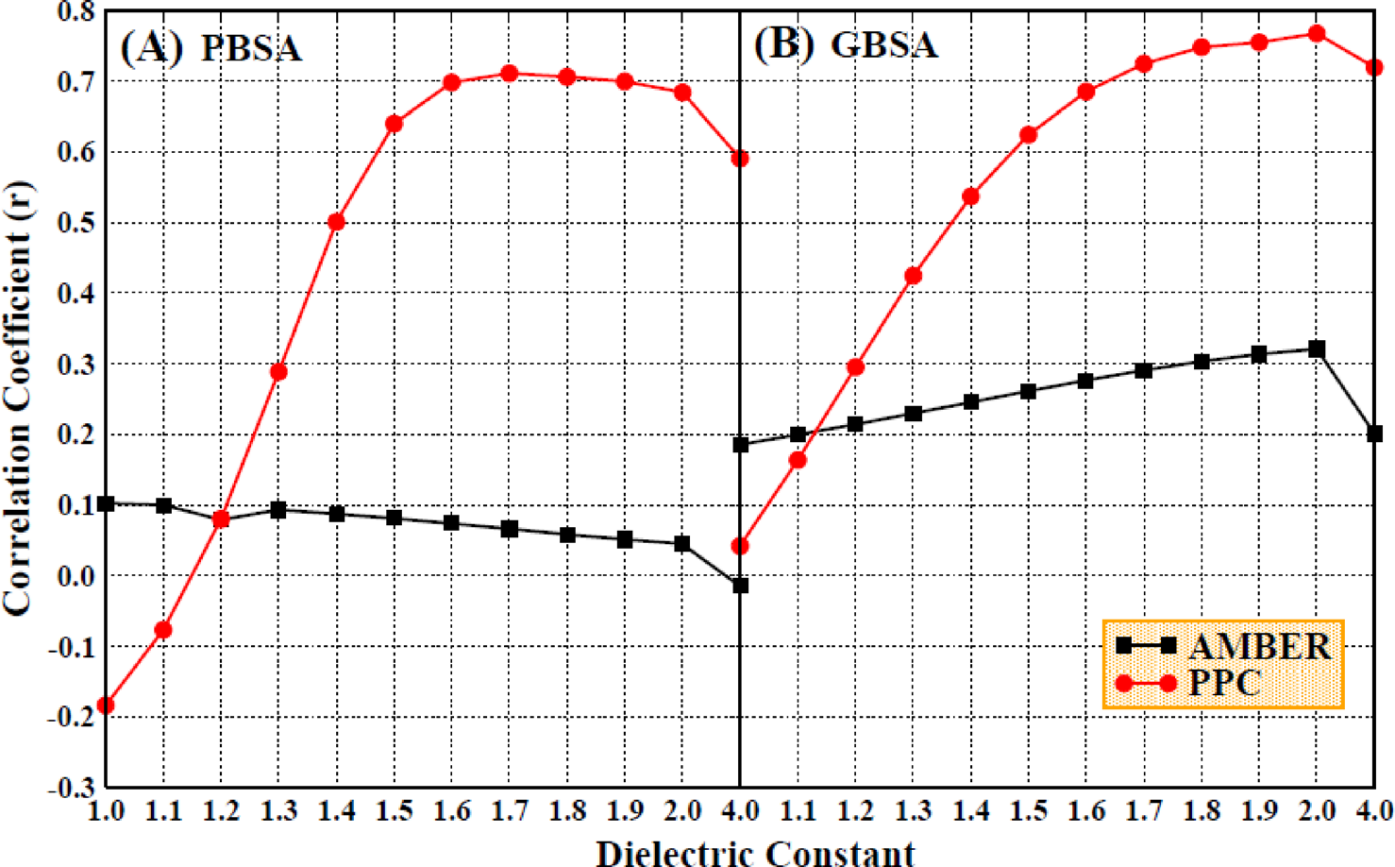
The correlation coefficients of enthalpy changes with experimental values under the AMBER and PPC force field (setting the interior dielectric constant to 1–2 with the interval of 0.1 and 4 for the electrostatic energy and 1 for polar solvation energy computation, (a) the left used the MM/PBSA method and (b) the right used the MM/GBSA method).

First, we examined the impact of a higher interior dielectric constant on the binding free energy when considering only the enthalpic contribution, without including the entropic change. The correlations between the theoretical calculations and the experimental results are shown in Fig. 8(a), which shows that the correlation coefficients are remarkably improved upon the increase in the interior dielectric constant (1.0–1.7) in the PPC force field. Compared to the results in the AMBER force field, the correlations are greater in the PPC force field, with the highest correlation coefficient (0.7) and slope (0.63) obtained with a dielectric constant of 1.7.

**FIG. 9.**
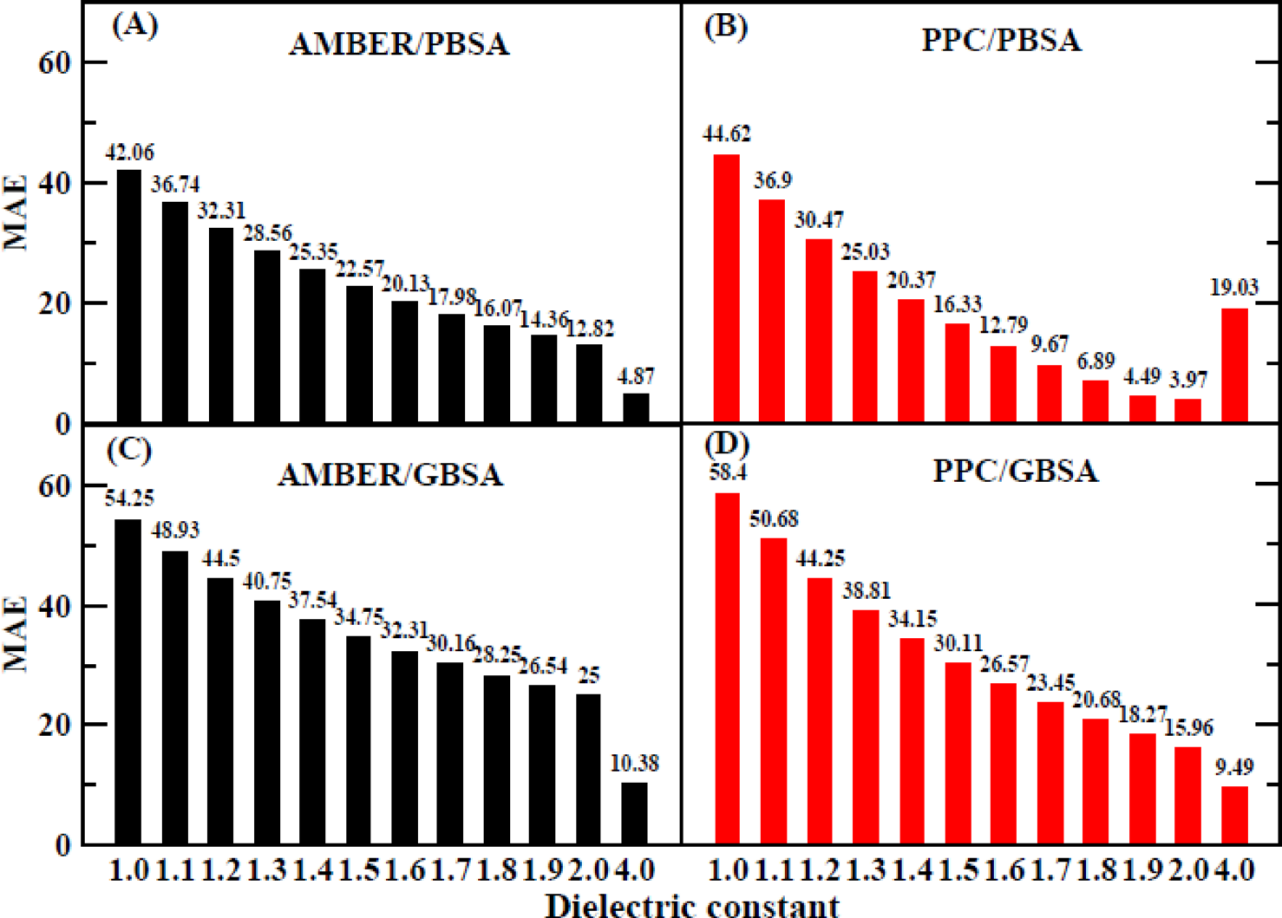
The MAE of enthalpy changes calculated by the MM/PB(GB) method under the AMBER and PPC force field. (a) AMBER/PBSA method, (b) PPC/PBSA method, (c) AMBER/GBSA method, and (d) PPC/GBSA method.

The mean absolute error (MAE) of the enthalpic change using the MM/PBSA method is displayed in Fig. 9. The lowest MAE is approximately 3.97 kcal/mol with an interior dielectric constant of 2.0 in the PPC force field, and it is 4.87 kcal/mol with an interior dielectric constant of 4.0 in the AMBER force field. Although the degree of correlation is somewhat improved, there are still great differences between the calculated and experimental values based on the above-mentioned MAE analysis.

**FIG. 10.**
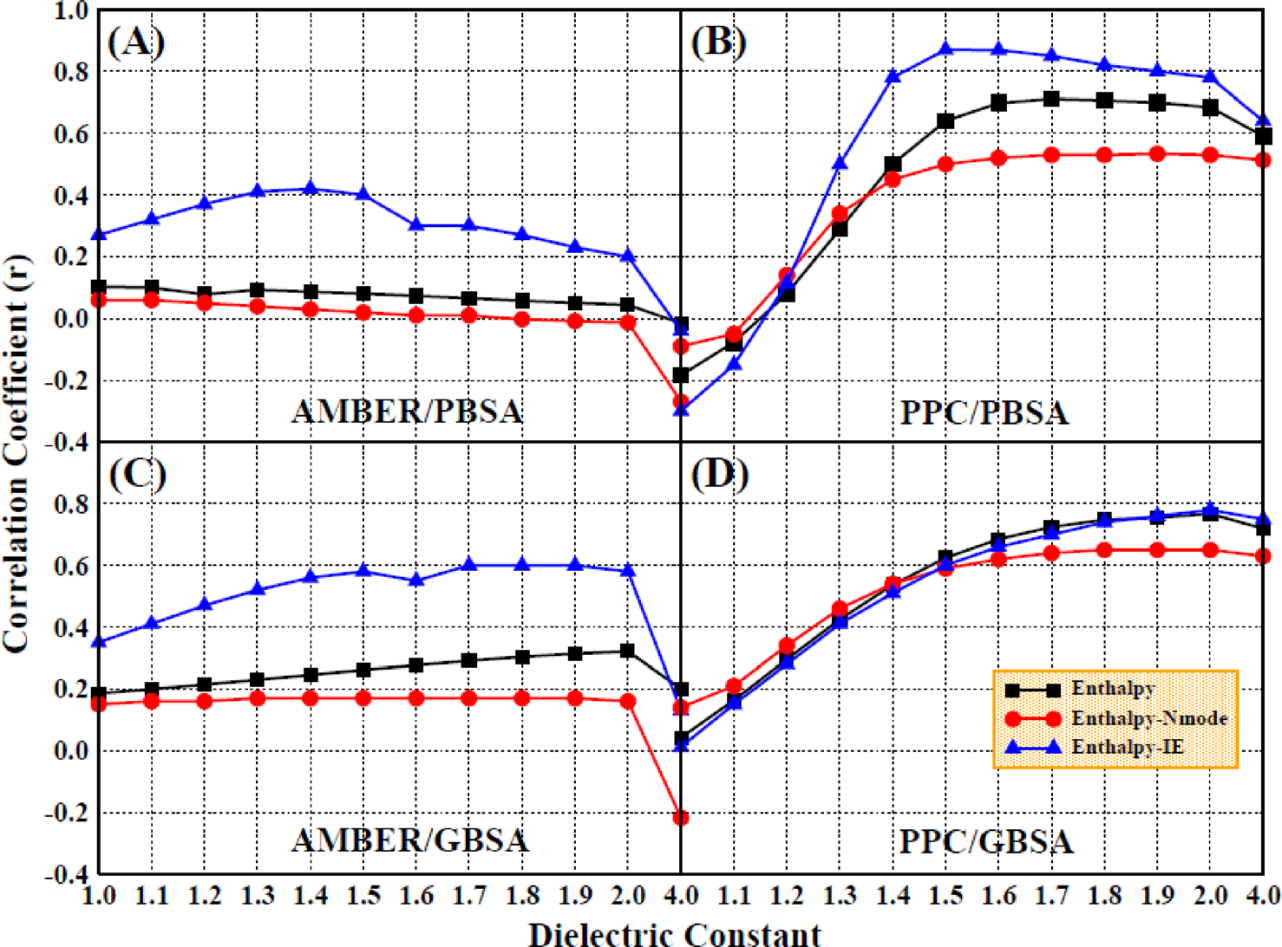
The same as Fig. 6, but adding the entropic contribution computed by the Nmode/IE method. (a) AMBER/PBSA method, (b) PPC/PBSA method, (c) AMBER/GBSA method, and (d) PPC/GBSA method.

Next, we once again included the entropic change in the calculation of the binding free energy. There were four different combinations of conditions used that included the two force fields used for MD simulation and the two methods used for the calculation of the entropic change, denoted AMBER/Nmode, AMBER/IE, PPC/Nmode, and PPC/IE. The results are shown in Figs. 10(a) and 10(b) for the different dielectric constants, which show that the correlation coefficients were significantly improved when the entropic contribution was computed using the IE method in either of the force fields. In addition, the correlation coefficients are decreased when using the Nmode method, which suggests that the entropic contribution calculated by the IE method is more accurate than that calculated by the Nmode method. The PPC/IE simulations produce higher correlation coefficients than the other three types of simulations, as shown in Figs. 10(a) and 10(b); the highest correlation coefficient, 0.87, is obtained when using an interior dielectric constant of 1.5. When the interior dielectric constant is 1.5–2.0 with an interval of 0.1, the value of the correlation coefficient is generally stable; when it is between 0.78 and 0.87, the coefficient fluctuated, and it decreases when the interior dielectric constant is 2.0–4.0.

Based on the above analysis, the PPC force field is found to be more reliable than the AMBER force field, and the IE method is also verified to be more accurate than the Nmode method. PPC/IE is the most suitable combination for the study of the HIV-1 protease. Therefore, the subsequent analysis utilizes the computational results of PPC/IE simulations.

**FIG. 11.**
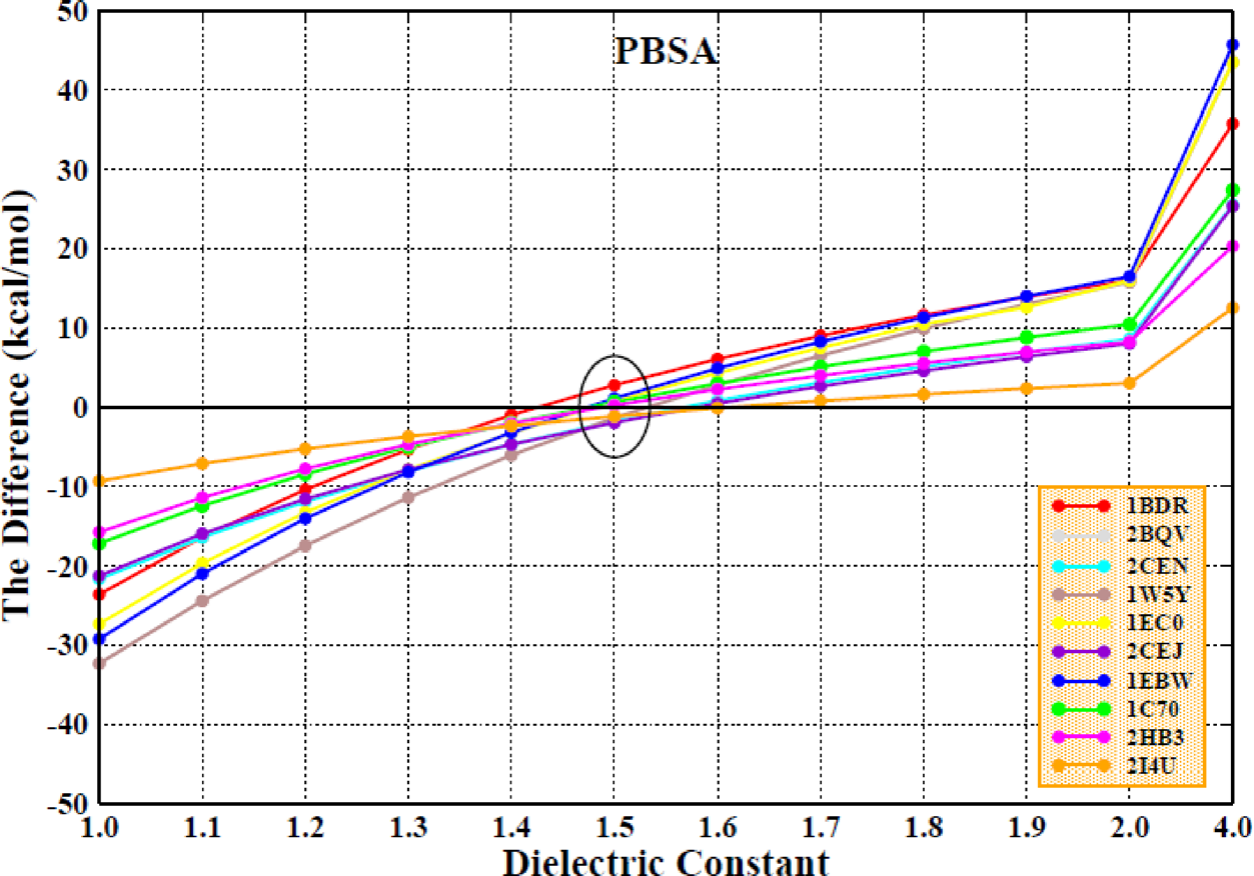
The differences between the calculated values and the experimental values of all ten systems in the PPC/IE combination and MM/PBSA method.

The differences in the binding free energy (ΔΔG) obtained from the calculated and experimental values for all ten systems in the PPC/IE force field are displayed in Fig. 11. It can be seen that the ΔΔG value for each of the ten systems is consistently close to zero when the interior dielectric constant is 1.5, which indicates that the calculated values are closest to the experimental values. Table I shows the total results and various terms for the calculation of the binding free energies for the 10 protease-inhibitor complexes in the PPC/IE force field with an interior dielectric constant of 1.5. As we noted previously, the differences in the binding free energies based on the calculated and experimental values are within 2 kcal/mol.

**TABLE I.** Binding free energy between HIV-1 protease and inhibitor in the combination of the PPC/IE method and the interior dielectric constant of 1.5 for 10 systems.

PDB code	$\Delta E_{ele}$	$\Delta E_{vdw}$	$\left\langle E_{pl}^{\mathrm{int}}\right\rangle$	$\Delta \Delta G_{pb}$	$\Delta \Delta G_{np}$	$\Delta \Delta G_{sol}$	ΔH	−TΔS	$\Delta G_{\text{bind}}$	$\Delta G_{\;\text{exp}\;}$
1BDR	−58.96	−60.50	−119.46	100.49	−7.37	93.12	−26.34	19.92	−6.42	−9.22
2BQV	−46.34	−69.85	−116.19	101.06	−7.66	93.40	−22.79	12.33	−10.47	−11.11
2CEN	−51.26	−76.19	−127.45	107.34	−8.42	98.92	−28.53	15.32	−13.21	−11.47
1W5Y	−73.98	−81.85	−155.83	136.33	−8.25	128.08	−27.75	14.77	−12.99	−11.64
1EC0	−72.21	−82.18	−154.39	135.66	−8.21	127.45	−26.94	16.01	−10.93	−11.73
2CEJ	−48.06	−74.49	−122.56	98.70	−7.95	90.75	−31.81	17.97	−13.84	−11.90
1EBW	−83.73	−74.66	−158.39	134.94	−8.11	126.83	−31.57	20.21	−11.36	−12.49
1C70	−57.93	−74.64	−132.57	114.32	−8.63	105.69	−26.88	13.27	−13.61	−14.23
2HB3	−33.35	−68.78	−102.13	75.51	−7.40	68.11	−34.02	17.21	−16.81	−15.67
2I4U	−39.98	−68.54	−108.52	83.67	−7.24	76.43	−32.09	16.36	−15.73	−15.98

The MAE values for all the interior dielectric constants are displayed in Fig. 13(a); the lowest value of approximately 1.24 is obtained when using the PBSA method with an interior dielectric constant of 1.5. The computational error is significantly reduced by including the entropic contribution to the binding free energy calculation when compared to the MAE for the enthalpic contribution, for which the lowest value is 3.97 kcal/mol. The second and third lowest values are 2.83 and 3.18 kcal/mol and are obtained at interior dielectric constants of 1.6 and 1.4, respectively. The lowest Standard Deviation (STD) value shown in Fig. 13(c) is approximately 1.43 kcal/mol and was obtained at an interior dielectric constant of 1.5, and the second and third lowest values are 1.46 and 1.91 kcal/mol and are obtained at values of 1.4 and 1.6, respectively. The lowest Root Mean Square Error (RMSE) shown in Fig. 13(e), 1.43 kcal/mol, also occurs at an interior dielectric constant of 1.5. The second and third lowest values are 3.41 and 3.49 kcal/mol and are obtained at interior dielectric constants of 1.6 and 1.4, respectively. In addition, the correlation coefficients are 0.87, 0.86, and 0.78 at interior dielectric constants of 1.5, 1.6, and 1.4, respectively (Fig. 10).

By calculating the correlation coefficients and analyzing the three types of error estimation, it can be determined that the suitable range for the interior dielectric constant is 1.4–1.6 when using the MM/PBSA method to calculate the binding free energy of the HIV-1 complex in this study.

In short, the use of the PPC/IE method with a higher interior dielectric constant can significantly improve the reliability and accuracy of the binding free energy calculation. The MM/PBSA method is used similar to the MM/GBSA method to compute the binding free energy. It is necessary to examine the impact of the interior dielectric constant on the binding free energy value that is obtained using the MM/GBSA method.

#### 3. The impact of the higher interior dielectric constant with the electrostatic energy and entropic contribution on the binding free energy obtained using the MM/GBSA method

In order to choose a suitable interior dielectric constant for use with the GB module and compare the accuracy of calculations using the MM/GBSA method with that of the MM/PBSA method, the solvation energy was calculated once again using the MM/GBSA method. The correlation coefficients for the comparison of the calculated enthalpic changes and the experimental data are displayed in Fig. 8(b). The correlation coefficients are improved with the increase in the interior dielectric constants within a range of 1–2 and are much greater in the PPC force field than in the AMBER force field, which is consistent with the calculated results obtained from the MM/PBSA method.

The MAE values of the enthalpic contributions when using the MM/GBSA method are also shown in Figs. 9(c) and 9(d). The MAE values are quite large for both the AMBER and PPC force fields. Therefore, we once again chose to take the entropic contribution into account when calculating the binding free energy. These results are shown in Figs. 10(c) and 10(d), which show that the consideration of the entropic change when using the IE method can improve the degree of correlation when using either the MM/GBSA or MM/PBSA method. Further experiments show that the consideration of the entropic contribution during the calculation of the binding free energy remarkably decreases the differences between the calculated values and the experimental values. The PPC/IE force field has the highest correlation coefficients among all the combinations (AMBER/PBSA/Nmode, AMBER/PBSA/IE, PPC/PBSA/Nmode, PPC/PBSA/IE, AMBER/GBSA/Nmode, AMBER/GBSA/IE, PPC/GBSA/Nmode, and PPC/GBSA/IE). The correlation coefficient is nearly converged when an interior dielectric constant from 1.7 to 2.0 is used with the GBSA method regardless of whether it is paired with AMBER/IE or PPC/IE, and the highest value of 0.78 is obtained in the PPC/IE simulation with a dielectric constant of 2.0.

**FIG. 12.**
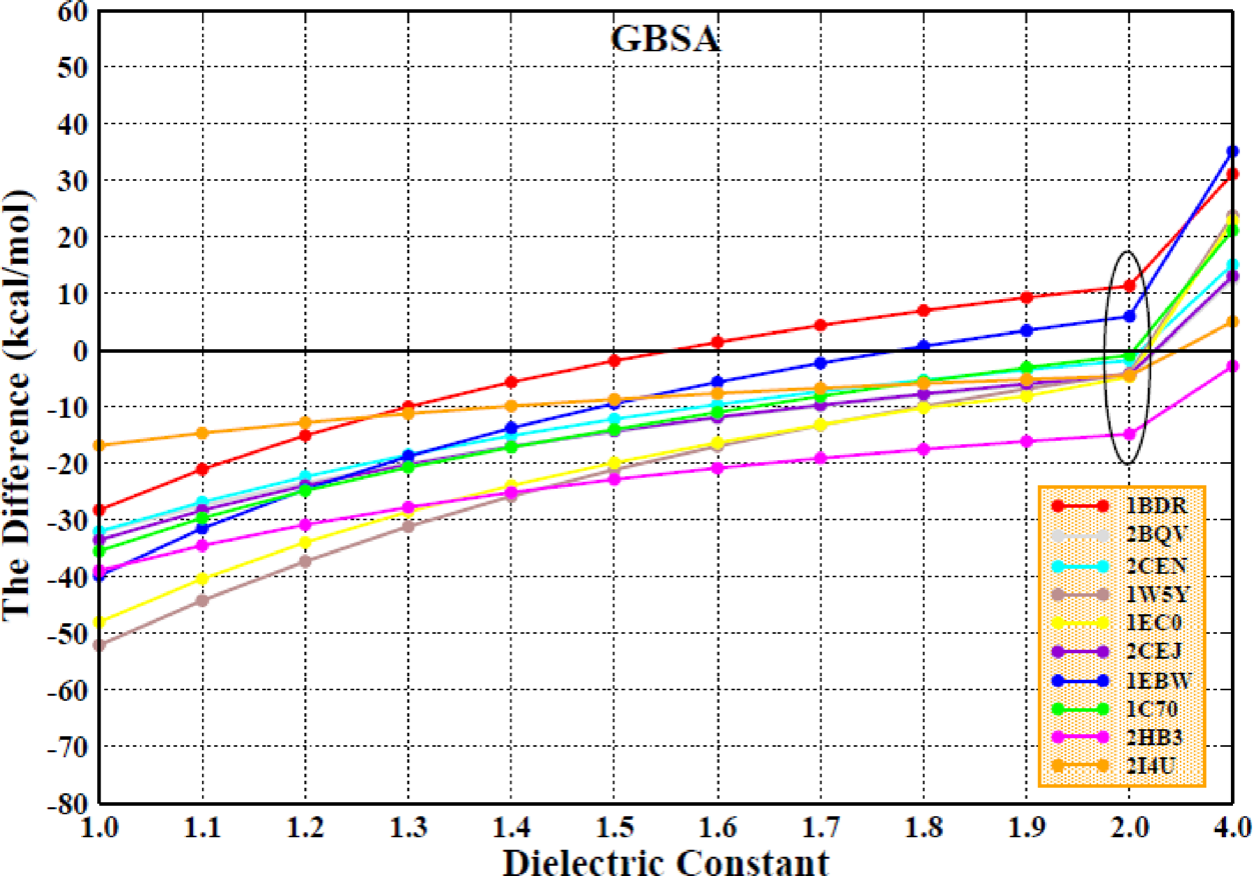
The same as Fig. 9, but for the MM/GBSA method.

The differences in the binding free energy (ΔΔG) obtained for the calculated and the experimental values for all ten systems when PPC/IE is combined with the MM/GBSA method are displayed in Fig. 12. When the interior dielectric constant is set to 2.0, a majority of the differences are close to zero, which indicates that those calculated values are in agreement with the experimental values.

**FIG. 13.**
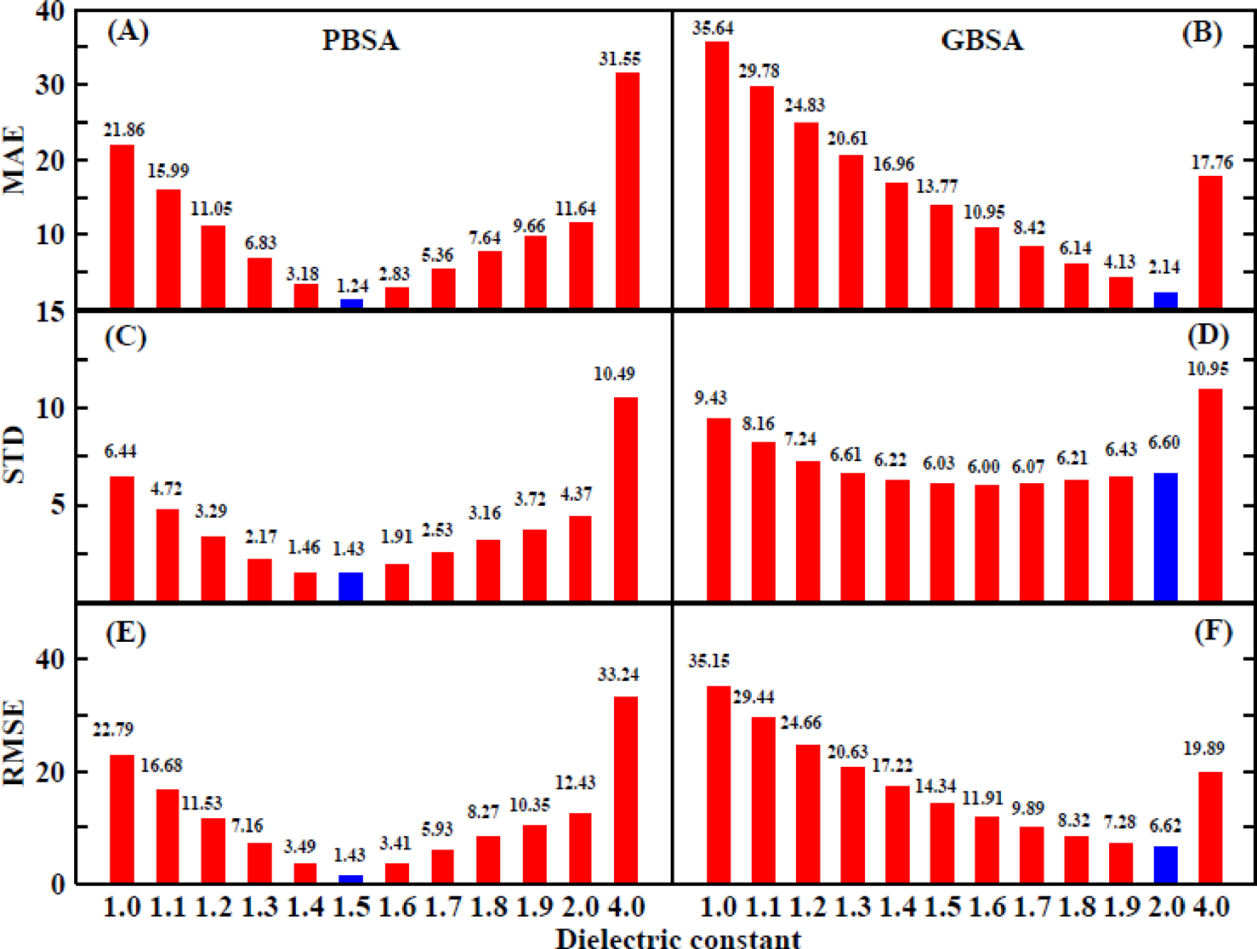
The MAE, STD, and RMSE between the calculated values and the experimental values of all ten systems in PPC/IE combination. (a) MAE, (c) STD, and (e) RMSE in the MM/PBSA method; (b) MAE, (d) STD, and (f) RMSE in the MM/GBSA method.

The MAE values for all the interior dielectric constants are displayed in Fig. 13(b); the lowest value, 2.14 kcal/mol, is obtained at an interior dielectric constant of 2.0 when using the GBSA method, which is consistent with a previous analysis. The values of MAE that are obtained when the entropic change is included in the calculation of the binding free energy are much smaller than those obtained when the entropic change is excluded (Fig. 9). The second and third lowest values are 4.13 and 6.14 kcal/mol and are obtained at interior dielectric constants of 1.9 and 1.8, respectively. When the interior dielectric constant ranged from 1.8 to 2.0, the values of the STD and RMSE are also relatively low, as shown in Figs. 13(d) and 13(f). We further examined the correlation between the calculated values of the interior dielectric constant in the range of 2–3 with an interval of 0.2 and the experimental value, which is displayed in Table S2. It can be found that the correlation had reached the highest (0.78) at 2.0 and the correlation was declining with the increase in the interior dielectric constants. Based on the previous analysis, the suitable range for the interior dielectric constant is 1.8–2.0 when using the MM/GBSA method.

Importantly, based on the MAE, STD, or RMSE values, the result obtained when using the PB module is far lower than that obtained from the GB module. This suggests that the PB module is more stable and reliable for use in the analysis of HIV-1 protease systems.

In summary, higher interior dielectric constants and entropic contribution values can improve the correlation coefficients obtained from the comparison of calculated and experimental values using the MM/PBSA and MM/GBSA methods, respectively. However, the MM/GBSA method is not as accurate as the MM/PBSA method in this study. The results also show that the PPC force field is more accurate than the AMBER force field and that the IE method is more rigorous than the Nmode method.

#### 4. Examination of the performance of the various methods

In order to verify the universality of our conclusions for the HIV-1 system, four additional HIV-1 protease systems with uncharged inhibitors (PDB entry: 3NU5, 1HPV, 3NUJ, and 3NUO) were re-analyzed using the same method. The binding free energy values, the correlation coefficients, and the MAE values are listed in Tables S3 and S4. Using the MM/PBSA method, the highest correlation coefficient obtained is 0.89 at an interior dielectric constant of 1.5–1.6, and the lowest MAE value is 2.85 kcal/mol at an interior dielectric constant of 1.4. The suitable range of the interior dielectric constant for the MM/PBSA method is still approximately 1.4–1.6. Using the MM/GBSA method, the lowest MAE value obtained is 5.34 and the highest correlation coefficient obtained is 0.97 at an interior dielectric constant of 1.8, which is within the optimal range that was given previously. The result of this test is in agreement with the previous described study and, to some extent, indicates that our analysis is credible to HIV-1 systems.

### C. The mechanisms underlying the interaction of HIV-1 and its inhibitors

In order to further examine the binding mechanisms utilized by the protease and its inhibitors, the contribution of every individual residue to the binding free energy was calculated at an interior dielectric constant of 1.5 using the MM/PBSA method combined with PPC/IE during the current study. For this analysis, it is helpful to predict the hot-spot residues within the protein. The favorable interactions that contributed to the binding free energy in each of the ten systems were decomposed into residue-inhibitor pairs in order to generate the residue-inhibitor interaction spectrum. During a previous study, it was found to be difficult to calculate the entropic contribution of each individual residue using the traditional Nmode method. Fortunately, one of the advantages of the IE method is that it can easily calculate the entropic contribution of each individual residue, which may improve the accuracy of the prediction of the hot-spot residues within the protease.

**FIG. 14.**
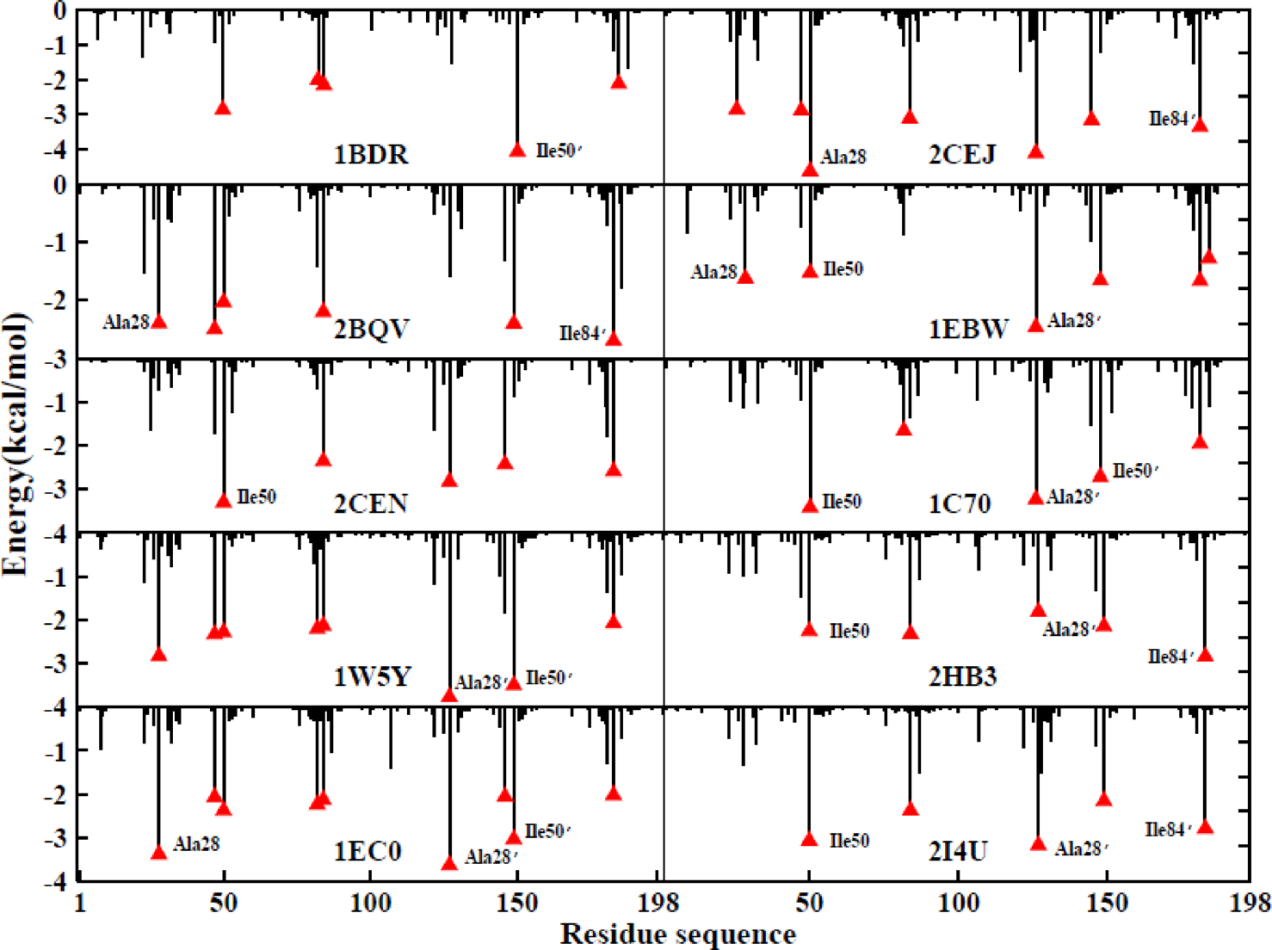
Decomposition of the binding free energy on a per-residue basis for all the ten systems in the PPC/PBSA/IE method.

Figure 14 shows the residue-inhibitor interaction spectra of the ten systems. The major contributors to the binding free energy appear to be the interactions of Ala28, Ile47, Ile50, Ala28′, Ile50′, and Ile84′ with the inhibitor. Among all the systems, the contribution of the A chain is nearly identical to that of the B chain, and the major residues that interact with the inhibitor are also similar. However, the contributions of the major residues in different systems lead to varying levels of performance.

For the 1BDR system, the Ile50′-inhibitor group makes the greatest contribution to the binding free energy (approximately −4.5 kcal/mol), and the contributions of the other groups are similar. As for the 2BQV and 2CEN systems, the Ile84′-inhibitor group makes the greatest contribution to the binding free energy of the 2BQV system, while the Ile50-inhibitor contributes most to that of the 2CEJ system. For the 1W5Y and IEC0 systems, the major residues are similar and the hot-spots are located at Ala28, Ala28′, and Ile50′; these residues contribute more than 2 kcal/mol to the binding free energy. For the 2CEJ system, the Ile28-inhibitor contributes the most to the binding free energy (approximately −4 kcal/mol), more than any other group. As for the 1EBW and 1C70 systems, their hot-spot residues are Ile50 and Ala28′. For the 2HB3 and 2I4U systems, the contribution of the major groups is similar, and the contribution of the Ile84′-inhibitor group within the 2I4U system is slightly greater than that in the 2HB3 system. This indicates that the major binding sites between all ten of the inhibitors and the HIV-1 protease are similar, and the differences in the affinity of individual residues for different inhibitors make little difference. Our studies may provide significant theoretical guidance for the rational design of drugs to inhibit the HIV-1 protease system.

### D. The contribution of the bridging water molecule to the binding free energy

Baldwin *et al.* first observed the four bridging water molecules within the HIV-1 protease-KNI-272 system and suggested the importance of these molecules for the design of potent inhibitors.^88^ Subsequently, the four water molecules were confirmed by Nuclear Magnetic Resonance (NMR) spectroscopy not to be an artifact resulting from crystallization.^89^ Subsequently, Wang *et al.* employed a double-decoupling free energy molecular dynamics simulation method to calculate the contribution of each of the four water molecules to the binding free energy,^90^ which found that WAT301 contributed significantly to the binding free energy. Due to the importance of the bridging water molecule WAT301 in the mechanism of interaction of the HIV-1 complex, it is necessary to investigate the impact of the WAT301 on the binding free energy.

**FIG. 15.**
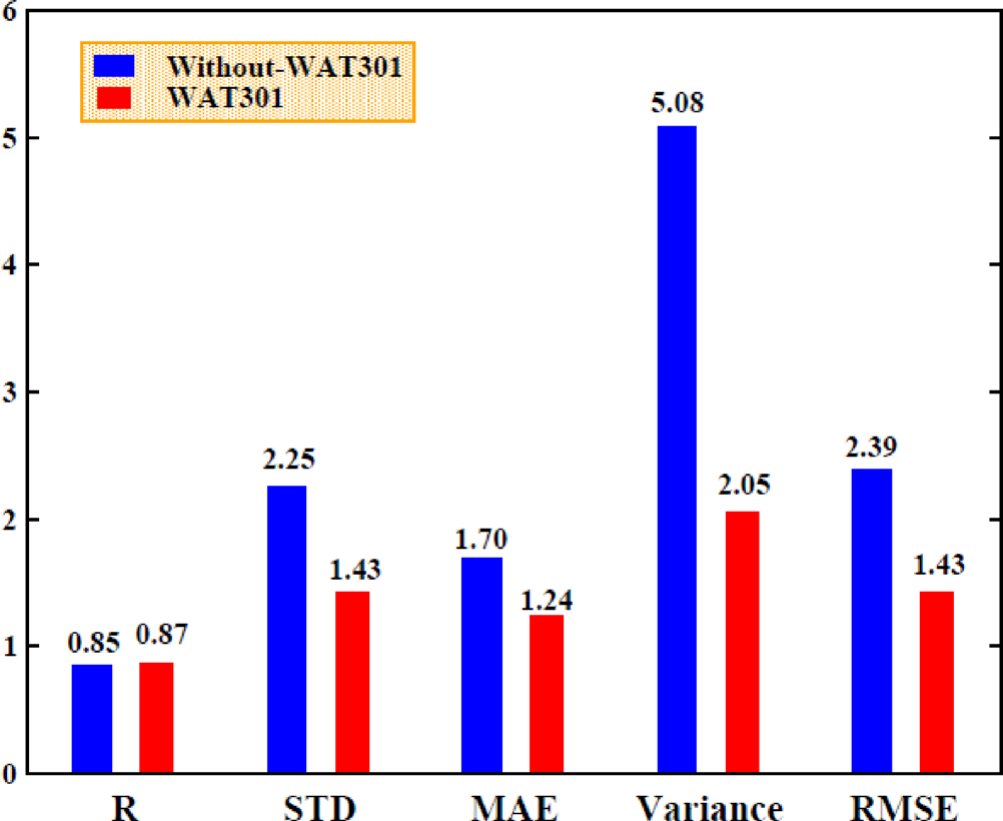
The comparison between the computed binding free energy (with WAT301 and without WAT301) and experimental values for correlation coefficients (R), STD, MAE, Variance (V), and RMSE in the PPC/PBSA/IE method.

The binding free energy in the absence of the WAT301 molecule was calculated using the MM/PBSA method in combination with PPC/IE with an interior dielectric constant of 1.5 for the electrostatic energy computation and an interior dielectric constant of 1 for the polar solvation energy computation. The correlation coefficients obtained from the comparison of the calculated values with the experimental values and the differences between them were estimated. The error analysis, which utilized the calculation of the STD, MAE, variance, and RMSE, was also carried out. The effects of WAT301 can be determined by comparing the binding free energy results obtained with and without the contribution of WAT301, as shown in Fig. 15.

Figure 15 shows the correlation coefficient obtained from the comparison of the calculated binding free energy with the contribution of WAT301 and the experimental result is approximately 0.87, while the correlation coefficient obtained without WAT301 is 0.85. It can be seen that the correlation coefficient is slightly greater with WAT301 than without WAT301, which indicates that the contribution of WAT301 is favorable to the affinity. The STD values of the binding free energy obtained with and without WAT301 are 1.43 and 2.25, respectively, while the MAE values obtained for the comparison of the calculated values and the experimental values are approximately 1.24 kcal/mol with WAT301 and approximately 1.70 kcal/mol without WAT301. The variance and RMSE values were also calculated to determine the effects of WAT301 and are both lower with WAT301 than without WAT301. The above result indicates that the calculated values are extremely consistent with the experimental values when the contribution of WAT301 is included in the prediction of the binding free energy, which demonstrates that including the effects of WAT301 can clearly improve the accuracy and reliability of the prediction of the affinity.

## IV. CONCLUSION

In this study, 10 ns MD simulations were carried out in both nonpolarized (AMBER) and polarized (PPC) force fields to investigate the impact of higher interior dielectric constants when using the MM/PB(GB)SA method to calculate the binding free energy of HIV-1 protease-inhibitor complexes. Simultaneously, the IE method was employed to calculate the entropic contribution to the binding free energy, which produced more accurate and reliable results than the traditional Nmode method. Our results can be summarized as follows:

First, the analysis of stability based on the values of the RMSD and interaction energy between the protease and the inhibitor demonstrated that the simulations were able to reach equilibrium in both force fields, which suggests that the PPC force field can provide more stable and reliable predictions of electrostatic interactions than the AMBER force field.

Second, the simultaneous increase in the interior dielectric constant for the calculation of both the electrostatic energy and the polar solvation energy did not result in good performance during our study. The reason may be that the electrostatic interaction is too strong in the HIV system. Therefore, the interior dielectric constant (1–2 with intervals of 0.1 and 4) was then adjusted only for the electrostatic energy calculation in order to search for an acceptable result for the binding free energy prediction. Based on the performance of the simulation as measured by the correlation between the experimental and theoretical values as well as the determination of three types of error estimation (MAE, STD, and RMSE), the suitable range for the interior dielectric constant is 1.4–1.6 for the MM/PBSA method and 1.8–2.0 for the MM/GBSA method. The MM/PBSA method was found to perform better than the MM/GBSA method in the calculation of the binding free energy for the HIV-1 protease system in the conditions produced by the use of the above-given values for the interior dielectric constant. The conclusion is only obtained from the HIV system, which is effective for further study on HIV protease. According to the above analysis, in some systems with strong electrostatic energy, higher dielectric for only electrostatic energy may lead to better results.

Third, the accuracy of the predicted binding free energy was clearly improved when using the PPC force field rather than the AMBER force field. In addition, the inclusion of the entropic change in the binding free energy prediction can significantly reduce computational errors, especially when using the IE method.

Fourth, decomposition of the residues was determined using the MM/PBSA method combined with the IE method, which was used to calculate the effects of entropic contribution on hot-spot residues. Although the mechanisms underlying the interactions within different complexes were not completely identical, the Ala28, Ile47, Ile50 Ala28′, Ile50′, and Ile84′ residues provide most of the energetically favorable contribution to the binding free energy within the ten complexes.

Fifth, the contribution of the bridging water molecule, WAT301, to the binding free energy was predicted using the MM/PBSA/IE method. In the calculation of the binding free energy, the explicit inclusion of the effects of WAT301 improves the accuracy of the calculated result.

We hope that the current study will provide essential guidance to the theoretical design and calculation methods used to generate new inhibitors targeting HIV-1 proteases.

## SUPPLEMENTARY MATERIAL

See supplementary material for the STD of entropy changes, the binding free energy, correlation coefficient (R), and MAE of the four systems in the optimal method, the fluctuation of interaction energy and entropy, and the hydrogen bond distance.
